# Retrait endoscopique d’un dispositif intra-utérin perforant le côlon sigmoïde: à propos d’un cas

**DOI:** 10.11604/pamj.2022.42.175.35808

**Published:** 2022-07-05

**Authors:** Cyrine Makni, Salma Souissi, Ahmed Saidani, Anis Belhaj, Olfa Bousnina, Leila Belhaj Ammar, Imen Ridene, Faouzi Chebbi, Lamia Kallel

**Affiliations:** 1 Service d'Hépato-gastro-entérologie, Hôpital Mahmoud El Matri, Ariana, Université de Tunis El Manar, Tunis, Tunisie,; 2 Service de Chirurgie Générale, Hôpital Mahmoud El Matri, Ariana, Université de Tunis El Manar, Tunis, Tunisie,; 3 Service de Radiologie, Hôpital Mahmoud El Matri, Ariana, Université de Tunis El Manar, Tunis, Tunisie

**Keywords:** Dispositif intra-utérin (DIU), perforation utérine, perforation du côlon sigmoïde, cas clinique, Intrauterine device (IUD), uterine perforation, sigmoid colon perforation, case report

## Abstract

Le dispositif intra-utérin (DIU) reste le pilier principal des mesures de planification familiale dans les pays en développement, néanmoins il est associé à des complications graves telles que les saignements, les perforations et les migrations vers des organes adjacents. Bien que la perforation de l'utérus par un DIU ne soit pas rare, la migration vers le côlon sigmoïde est exceptionnelle. Nous rapportons ici un cas de migration d'un stérilet vers le côlon sigmoïde qui a été retiré par voie endoscopique basse. Il s´agit d´une femme de 45 ans, porteuse d'un stérilet, se présente 6 ans plus tard, avec des douleurs pelviennes à type de pesanteur. L´examen clinique était sans anomalies et l´exploration scannographique avait objectivé le DIU qui était incrusté dans la paroi du colon sigmoïde. Une laparoscopie à visée diagnostique et thérapeutique a été réalisée; elle avait objectivé une perforation intestinale par le dispositif, qui était partiellement incrusté dans le côlon sigmoïde, mais elle avait échoué de l´extraire. Le dispositif avait été retiré lors d´une coloscopie par une anse diathermique (15mm de diamètre).

## Abstract (en)

Intrauterine device (IUD) is the mainstay of family planning methods in developing countries. However, it is associated with severe complications such as bleeding, perforation and migration to adjacent organs. Although perforation of the uterus is not rare, migration to the sigmoid colon is exceptional. We here report a case of IUD migration into sigmoid colon; this was removed via low endoscopy. The study involved a 45-year-old woman using an IUD who presented with pelvic pain associated with a feeling of pelvic heaviness 6 years later of insertion. Clinical examination was without abnormalities, and computed tomography (CT) scan showed the IUD embedded in the sigmoid colon wall. Diagnostic and therapeutic laparoscopy was performed, which objectified IUD-related intestinal perforation. IUD was partially embedded in the sigmoid colon wall and couldn’t be removed. The device was removed during colonoscopy by diathermy loop excision (15 mm in diameter).

## Introduction

Le dispositif intra-utérin (DIU) constitue l'un des moyens contraceptifs les plus utilisés au monde: 9,4% dans les pays développés et 16,5% dans les pays non développés [1]. C'est une méthode de contraception simple, efficace, globalement bien tolérée et peu couteuse. Le DIU permet d'assurer une contraception de longue durée sans poser le problème de l'observance. Cependant, ses complications doivent être connues pour bien optimiser son action. La perforation utérine est l'une des complications les plus rares, avec une incidence entre 1,3 et 1,6 pour 1000 insertions [2], et les plus graves, qui peuvent engendrer la migration du DIU dans les différents organes de voisinage tel que le mésentère, le colon et la vessie. Nous rapportons un cas de migration du DIU dans la cavité péritonéale avec pénétration dans le colon sigmoïde, dont le diagnostic a été retenu six ans après son insertion, et ce dans le cadre de l´exploration de douleurs pelviennes. L'abdomen sans preparation et la tomodensitométrie étaient les moyens de diagnostic de cette migration. La coloscopie avait permis de retirer le DIU, après échec d´une tentative d´extraction chirurgicale par voie laparoscopique.

## Patient et observation

**Information de la patiente:** nous rapportons le cas d´une femme, âgée de 45 ans, multipare, sans antécédents pathologiques notables, ayant eu ses accouchements par voie basse et connue porteuse d'un dispositif intra-utérin T depuis 2013. Six ans après, la patiente a consulté aux urgences devant la survenue de douleurs pelviennes à type pesanteur, sans signes digestifs associés.

**Résultat clinique:** l´examen abdominal avait objectivé une légère sensibilité de la fosse iliaque gauche. Les touchers pelviens étaient sans anomalies. L´examen gynécologique n'avait pas mis en évidence le fils du stérilet en intra-vaginal.

**Démarche diagnostique:** la patiente avait bénéficié d´une échographie pelvienne qui n´avait pas retrouvé le stérilet en intra-utérin. Un scanner abdominal avait été réalisé et avait montré que le DIU était partiellement incrusté dans la paroi du colon sigmoïde. Le diagnostic de perforation secondaire de l'utérus avec migration en intraabdominale et perforation du colon sigmoïde avait été retenu.

**Figure 1:**
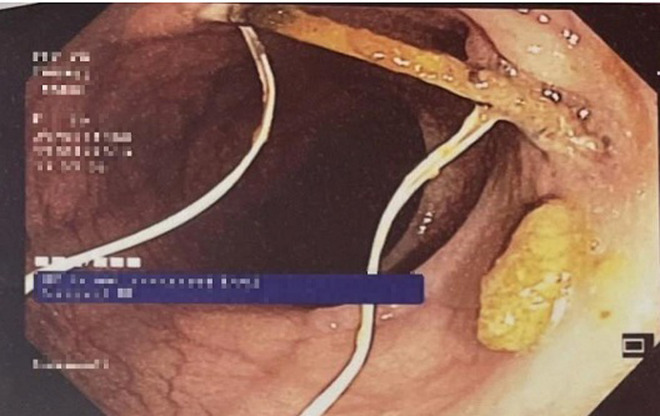
image coloscopique du DIU perforant le colon sigmoïde et s’incrustant dans sa paroi

**Figure 2:**
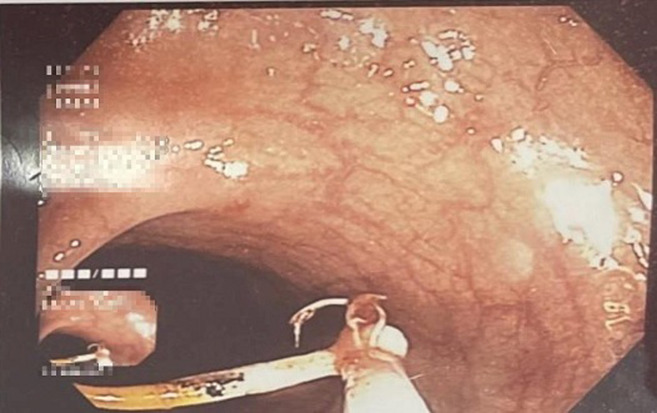
retrait du DIU perforant le colon par voie coloscopique à l’anse diathermique (15mm)

**Figure 3:**
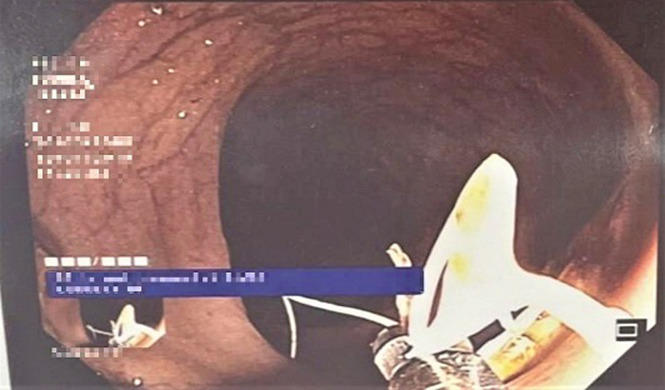
DIU complètement retiré de la paroi du colon sigmoïde

**Intervention thérapeutique et suivi:** une laparoscopie à visée thérapeutique avait été indiquée pour la patiente; elle avait confirmé la présence du DIU qui perfore le colon sigmoïde, cependant la tentative de son extraction avait échouée. Une tentative d´extraction par voie endoscopique a été proposée. La coloscopie avait objectivé un corps étranger localisé à 20 cm de la marge anale et correspondant au dispositif intra-utérin (Figure 1). Il était partiellement incrusté dans la paroi colique avec un aspect surélevé et inflammatoire de la muqueuse adjacente. Le DIU avait pu être désinséré et retiré de la lumière colique avec l´anse diathermique (15mm) Figure 2, Figure 3. La patiente avait été revue un mois puis 3 mois après, elle était bien portante avec disparition des douleurs pelviennes.

**Consentement du patient:** un consentement éclairé de la patiente avait été obtenu.

## Discussion

Le dispositif intra-utérin est une méthode de contraception largement utilisée, préférée en raison de la longue durée du contrôle des naissances et de la facilité d'utilisation. Cependant, c´est une méthode qui peut engendrer de sérieuses complications telles que les infections gynécologiques qui sont les plus fréquentes [3], puis surviennent les perforations utérines avec une incidence faible, ne dépassant pas 1,6 pour 1000 insertions [2]. La migration vers les structures abdomino-pelviennes est rare [4]. Les sites les plus courants de perforation intestinale sont le rectum, l'intestin grêle et le côlon sigmoïde [5], tel est le cas de notre patiente. Le dispositif peut être partiellement ou totalement encastré dans la paroi intestinale [5]. Les symptômes d´une perforation du DIU sont divers et variés, allant d´une grossesse non désirée ultérieure [6], jusqu´à des signes urinaires irritatifs [7], des douleurs pelviennes chroniques, une péritonite aiguë, des fistules et des abcès au niveau des organes de pénétration. L´échographie et la radiographie standard permettent de diagnostiquer un corps étranger échogène et radio-opaque, respectivement. La tomodensitométrie est une technique d'imagerie utile, comme dans notre cas, pour la confirmation de la localisation du stérilet [4].

L´Organisation mondiale de la Santé recommande la suppression d'un dispositif intra-utérin disloqué aussitôt que possible, quel que soit son type et son emplacement [8]. Il est conseillé de récupérer un DIU migré par des techniques mini-invasives [9]. Les techniques coloscopiques et laparoscopiques [2] sont de plus en plus utilisées pour le retrait des DIU. La coloscopie est utile dans les cas où le dispositif se trouve dans la lumière ou intégré dans la partie interne de la paroi. Cependant, la récupération coloscopique peut entraîner des difficultés si le dispositif est partiellement intégré dans des structures adjacentes et entouré de tissu de granulation. Cette intervention serait traumatique et pourrait provoquer une lésion colique avec fuite intra abdominal du contenu colique [2]. Une revue de techniques chirurgicales pour retirer le DIU [9], a révélé que, 93% des cas dans la littérature ont été retiré par laparoscopie, mais les cas de perforations des organes abdominaux et pelviens ont été retirés par laparotomie dans 57,1%. Dans notre cas, nous avons récupéré le stérilet par voie endoscopique basse après échec de l´extraction chirurgicale par voie laparoscopique.

## Conclusion

Le DIU est une méthode de contraception efficace cependant elle peut entrainer des complications graves. La perforation utérine associée à une migration intra intestinale est l'une des complications les plus rares et les plus redoutables. La coloscopie peut constituer une alternative efficace pour son ablation, tel est le cas de notre patiente.
